# 1848. Barriers and Facilitators to HIV Care in the Transgender Population

**DOI:** 10.1093/ofid/ofad500.1676

**Published:** 2023-11-27

**Authors:** Dora Martinez, Dana D Hines, Arianna Inurritegui-Lint, Kelly E Pillinger, Laura Simone, Leah Molloy

**Affiliations:** Viiv Healthcare, Harlingen, Texas; Health Resources Services Administration HIV/AIDS Bureau, Rockville, Maryland; Arianna’s Center, Fort Lauderdale, Florida; PRIME Education, LLC, New York, New York; PRIME Education, LLC, New York, New York; PRIME Education, LLC, New York, New York

## Abstract

**Background:**

Transgender-inclusive healthcare focused on HIV prevention and treatment is a critical component to ending the HIV epidemic. Understanding the needs of this population is essential.

**Methods:**

Between March – May 2022, surveys were distributed by Arianna’s Center to the transgender community to assess barriers and facilitators related to HIV care services. These results informed a national webinar for healthcare providers (HCPs), who were surveyed before and after the webinar.

**Results:**

There were 254 individuals who completed the survey (50% transgender female; 20% transgender male; 58% LatinX; 30% African American; 31% living with HIV). The biggest barriers to accessing or staying engaged in HIV prevention/care services were socioeconomic (24%), discrimination/racism/transphobia (15%), and mental health challenges (13%).

Over half (52%) felt there were barriers to initiating conversations about sexual health and HIV and 27% reported being denied healthcare services because of their gender and/or sexual orientation.

For those taking medication for HIV prevention or treatment (59%, n = 150/254), the biggest adherence facilitator was having a supportive provider knowledgeable about transgender health (56%, n = 84/150). However, there was a disconnect between those who felt highly involved in decisions regarding their sexual/HIV health and those who wanted to be highly involved (Fig. 1). Among those who felt uninvolved in these care decisions (54%, n = 137/254), 39% (n = 53/137) felt too overwhelmed with other things in their lives and 24% (n = 33/137) felt they tried to be involved but their care team did not listen.

Most HCPs (55%, N = 98/178) attending the webinar had little to no experience caring for transgender individuals. Only 12% (n = 21/178) of HCPs felt they included their transgender clients extremely well in their HIV care (Fig. 1). Notably, there was improvement in HCPs’ knowledge (p < .001) and confidence in counseling (p < .001) about drug interactions between antiretrovirals and gender-affirming hormone therapy.

**Figure:**
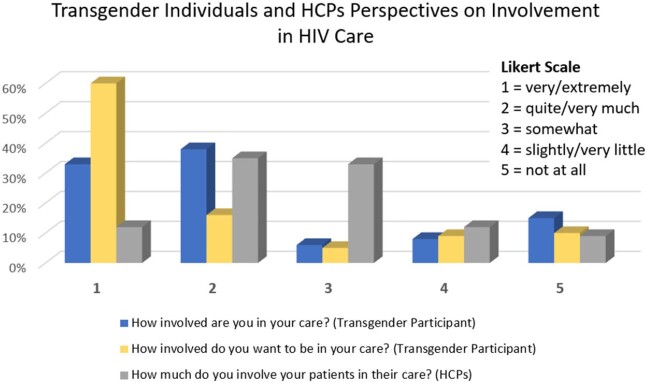

**Conclusion:**

Insights from the transgender community indicate potential areas for improvement in HIV care, and can be leveraged to support HCP education to improve engagement with transgender individuals and promote shared decision-making.

**Disclosures:**

**Dora Martinez, MD, FAAFP, AAHIVS**, Gilead Sciences: Advisor/Consultant|Gilead Sciences: Honoraria|Janssen: Honoraria|ViiV Healthcare: Advisor/Consultant|ViiV Healthcare: Honoraria|ViiV Healthcare: As of May 16th will be employed by ViiV, however was not a ViiV employee at time of project **Arianna Inurritegui-Lint, -**, Gilead Sciences, Inc.: Advisor/Consultant|Human Rights Campaign Foundation: Advisor/Consultant|National Minority AIDS Council: Advisor/Consultant|ViiV Healthcare: Advisor/Consultant

